# Clinical and Functional Characterization of Novel INSR Variants in Two Families With Severe Insulin Resistance Syndrome

**DOI:** 10.3389/fendo.2021.606964

**Published:** 2021-04-29

**Authors:** Qiaoli Zhou, Jing Yu, Xuewen Yuan, Chunli Wang, Ziyang Zhu, Aihua Zhang, Wei Gu

**Affiliations:** ^1^ Department of Endocrinology, Children’s Hospital of Nanjing Medical University, Nanjing, China; ^2^ Nanjing Key Laboratory of Pediatrics, Children’s Hospital of Nanjing Medical University, Nanjing, China; ^3^ Department of Nephrology, Children’s Hospital of Nanjing Medical University, Nanjing, China; ^4^ Jiangsu Key Laboratory of Pediatrics, Nanjing Medical University, Nanjing, China

**Keywords:** Donohue syndrome, type A insulin resistance, diabetes mellitus, hirsutism, insulin receptor gene, mutation

## Abstract

**Objective:**

Defects in the insulin receptor (*INSR*) gene cause various severe insulin resistance conditions, including Donohue syndrome (DS), Rabson-Mendenhall syndrome (RMS) and type A insulin resistance (type A-IR). This study aimed to investigate the clinical characterization and molecular defects in three Chinese children with *INSR*-related insulin resistance syndrome.

**Methods:**

We reviewed the clinical data of three Chinese children with *INSR*-related insulin resistance syndrome from two unrelated kindreds. Genetic analysis was performed using whole-exome sequencing and the effects of the novel variants were further assessed by *in vitro* functional assays.

**Results:**

The proband with type A-IR presented with acanthosis nigricans, hypertrichosis, and euglycemia with mild insulin resistance in early childhood. His sister presented with features typical of type A-IR and was diagnosed with diabetes mellitus with severe insulin resistance at the age of 9.8 years. The proband with DS showed typical dysmorphic characteristics, severe intrauterine growth retardation, extreme insulin resistance, fasting hypoglycemia and postprandial hyperglycemia from birth. The heterozygote variants c.[3670G>A]; c.[3614C>T] were identified in both siblings with type A-IR; and c.[749_751del]; c.[3355C>T] in the patient with DS. *In vitro* studies showed that the novel variant c.749_751del [p.(Thr250del)] in the α-subunit, reduced expression of the mature INSR protein and severely impaired INSR function. In contrast, the novel variant c.3670G>A [p.(Val1224Met)] in the β-subunit had no effect on total protein expression and phosphorylation of INSR and Akt, suggesting that the variant p.Val1224Met appeared to be tolerated and was not responsible for the severe insulin resistance.

**Conclusion:**

Our study detailed the clinical features of three patients with type A-IR and DS, and identified two novel variants in the *INSR* gene. Functional assays indicated the novel variant p.Thr250del was pathogenic. In contrast, the novel variant p.Val1224Met was suggested to be tolerated by our experimental data, even though bioinformatics analyses predicted the variant as deleterious.

## Introduction

Severe insulin resistance caused by loss-of-function mutations in the insulin receptor (*INSR*) gene comprises a wide phenotypic spectrum, including Donohue syndrome (DS), Rabson-Mendenhall syndrome (RMS) and type A insulin resistance (type A-IR) (1). DS (also known as leprechaunism), the most severe form of these syndromes, has a prevalence of approximately 1 in 4 million births (2). It is characterized by intrauterine and postnatal growth retardation, developmental delay, thick skin with lack of subcutaneous fat, hirsutism and characteristic facies, as well as severely abnormal glucose homeostasis and extreme insulin resistance. Patients with DS usually die within the second year of life. RMS is a less severe disease but also features insulin resistance, acanthosis nigricans, soft tissue overgrowth, coarse facial features and linear growth impairment (3). Type A-IR is relatively mild and is characterized by insulin resistance, acanthosis nigricans and hyperandrogenism that is usually identified peri- or post-pubertally (4). The wide diversity in the clinical presentation of *INSR*-related insulin resistance syndrome is generally correlated with the type and localization of the genetic defect in the *INSR* gene (5).

The *INSR* gene is located on chromosome 19p13.2-13.3 and includes 22 exons. It encodes a protein of 1382 amino acids that belongs to the Src family of tyrosine specific protein kinases (6). After being transported from the endoplasmic reticulum to the Golgi apparatus, the pre-protein is further glycosylated and then cleaved into a α2β2 heterotetramer, followed by transport to the plasma membrane. The extracellular α chains carry the insulin-binding regions, while the transmembrane β chains have intrinsic tyrosine kinase activity (7). The INSR protein is composed of the following domains: a leucine-rich repeat domain (L1), a cysteine-rich region (CR), a second leucine-rich repeat domain (L2), three fibronectin type III (FnIII) domains (FnIII-1 to FnIII-3) and a tyrosine kinase domain (TK) (8). Most patients with DS or RMS have biallelic variants in the INSR α-subunit, which decrease the binding affinity to insulin and/or impair INSR processing. A proportion of patients with type A-IR have heterozygous variants in the intracellular TK domain of the β-subunit, which affect kinase activity and lead to a mild decrease in insulin binding capacity (9, 10).

In this study, we investigated three patients in two unrelated kindreds with *INSR*-related insulin resistance syndrome, including one case of DS and two cases of type A-IR. Two novel variants (c.749_751del and c.3670G>A) in the *INSR* gene were identified by whole-exome sequencing. The novel variants were assessed for their effect on INSR function *in vitro* by expressing the mutant receptor in Chinese hamster ovary (CHO) cells, which have a low background of endogenous INSR.

## Materials and Methods

### Subjects

**Figure 1:**
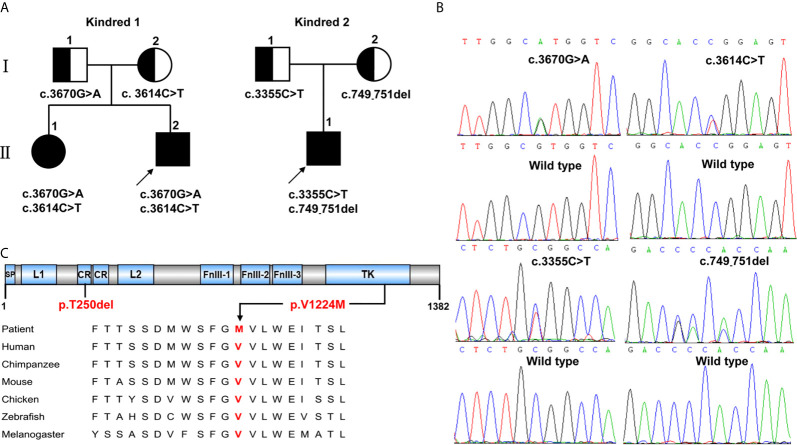
*INSR* variants identified in two kindreds with severe insulin resistance syndrome. **(A)** The kindreds of patients with *INSR*-related insulin resistance syndrome and *INSR* variants. Kindred 1 with type A-IR; Kindred 2 with DS. **(B)** Sanger sequencing analysis showing the *INSR* gene variants. **(C)** The positions of the *de novo* variants p.Thr250del (p.T250del) and p.Val1224Met (p.V1224M) identified in patients 1,II.2 and 2,II.1 are shown below the INSR domains. The valine residue at position 1224 is highly conserved across different species.

We investigated two Chinese kindreds with *INSR*-related insulin resistance syndrome, including two siblings with type A-IR in Kindred 1 and a proband with DS in Kindred 2 (**Figure 1A**). Parents of both probands were non-consanguineous, healthy and phenotypically normal, without any history of diabetes.

### Whole-Exome Sequencing and Bioinformatics Analysis

To identify the underlying genetic defect, whole-exome sequencing was performed on genomic DNA samples isolated from the peripheral blood. Sanger sequencing was performed to confirm the identified variants. Four types of software (Mutation Taster, Provean, PolyPhen-2 and SIFT) were used to predict the damaging effect of the novel variants on INSR function.

### Construction of Expression Plasmids

Wild-type (WT) full-length human *INSR* cDNA (NM_000208) was purchased and cloned into a pcDNA3.1-3xFlag vector using a Clone Express Entry one-step cloning kit (Vazyme Biotech Co., Ltd, China). The variants (c.749_751del and c.3670G>A) were introduced into the pcDNA3.1-Flag-tagged plasmid using site-directed mutagenesis according to the PCR-based DpnI-treatment method (Vazyme Biotech Co., Ltd, China). Targeted sequences were confirmed by dideoxy-based sequencing.

### Transient Transfection of CHO Cells

CHO cells were maintained in RPMI-1640 medium supplemented with 10% fetal bovine serum (FBS) (Gibco; Thermo Fisher Scientific, Inc.) at 37°C in an atmosphere of 5% CO_2_. After the cells were 50-70% confluent in 6- or 24-well plates, they were transfected with expression vectors containing *INSR* (wild or mutant type) using Lipofectamine 2000 (Invitrogen) according to the manufacturer’s protocols.

### Immunofluorescence Analysis

To determine the localization of INSR, CHO cells were seeded into 24-well culture plates containing glass slides in RPMI-1640 medium with 10% FBS. About 36 h after transfection, the cells were fixed with 4% paraformaldehyde for 15 min, following permeation with 0.2% Triton X-100. Next, the cells were incubated overnight at 4°C with primary mouse anti-Flag (1:400; Sigma-Aldrich, St. Louis, MO) and rabbit anti-Na^+^/K^+^-ATPase antibodies (1:400; Abcam, Cambridge, MA), followed by fluorescein-conjugated secondary antibodies for 2 h at 37°C. The cell nucleus was counterstained with 4’, 6-diamidino-2-phenylindole. Cells were imaged with an inverted confocal laser scanning microscope using a 63/1.4 oil-immersion objective.

### Western Blot Analysis

To evaluate the activation of INSR and its downstream signaling, total INSR, phospho-INSR and total Akt, phospho-Akt were measured by western blot with and without insulin stimulation. A Flag-tagged *INSR* expression vector (wild or mutant type) was transfected into CHO cells. For phosphorylation assays, CHO cells were serum-starved 48 h after transfection for 4 h, before stimulation with 10 nmol/L insulin or vehicle control for 5 min at 37°C. 40 μg of total protein was resolved by 10% SDS-PAGE before transfer to PVDF membranes. The membranes were then incubated with anti-Flag (1:3000, Sigma-Aldrich), anti-INSR (1:1000, Abcam), anti-phosphorylated INSR (1:1000, Abcam), anti-Akt (1:1000, Cell Signaling Technology), anti-phosphorylated Akt (1:1000, Cell Signaling Technology), or GAPDH (1:3000, ProTech), followed by secondary horseradish peroxidase-labeled antibody (1:3000, Beyotime, China). Membranes were visualized by chemiluminescence reaction. Band intensity was quantified using ImageJ software (National Institutes of Health, Bethesda, MD).

## Results

### Clinical Characteristics

The clinical features and laboratory data of the patients are summarized in **Table 1**. We followed the CARE guidelines (11). Patients’ photography cannot be presented in this research work being unable to have the consent from the patients, which is a limitation of current study. The patient’s perspective is shown in supplemental material.

**Table 1:** Clinical features and laboratory data of the three patients.

Patients	1,II.1	1,II.2	2,II.1
**Sex**	F	M	M
**Gestational age (w)**	40	40	37
**Birth-height (cm)**	ND	ND	43 (-2.26 SDS)
**Birth-weight (kg)**	3.3 (-0.23 SDS)	3.0 (-1.26 SDS)	1.6 (-3.33 SDS)
**Head circumference (cm)**	ND	ND	27
**Current age (years)**	9.8	3.5	2
**Height (cm)**	140.5 (+0.23 SDS)	92.5 (-2.12 SDS)	74.3 (-4.21 SDS)
**Weight (kg)**	27 (-0.81 SDS)	12 (-2.43 SDS)	8.4 (-3.6 SDS)
**Dysmorphic features**	–	–	+
**Acanthosis nigricans**	+	+	+
**Decreased subcutaneous fat**	–	–	+
**Fasting glucose (mmol/L)**	4.48	4.18	3.0
**Fasting insulin (mU/L)**	126.3	54.89	>1000
**Fasting C-peptide (nmol/L)**	1.66	0.65	>13.33
**2h postload glucose (mmol/L)**	15.98	5.43	15
**2h postload insulin (mU/L)**	464.2	295.30	>1000
**2h postload C-peptide (nmol/L)**	>13.33	2.16	>13.33
**HbA1c (%)**	7.1	5.5	ND

ND, not detertmined; SDS, standard deviation score.

Kindred 1 consisted of healthy parents and two affected children (**Figure 1A**). The proband (1, II.2) was born as the second child to non-consanguineous parents at 40 weeks of gestation with a birth weight of 3.0 kg (-1.26 SDS). At the age of 3.5 years, he was admitted to our department because of increased hyperpigmentation of the skin over the neck and axilla since he was 1.5 years old. On physical examination, his height was 92.5 cm (-2.12 SDS) and his weight was 12 kg (-2.43 SDS). Generalized hypertrichosis and skin pigmentation were observed, with acanthosis nigricans of the neck and axillae. He did not show the dysmorphic features characteristic of DS or RMS. Laboratory data revealed a normal fasting serum glucose level of 4.18 mmol/L [Normal range (NR), 4.1-5.9mmol/L)]; a normal postprandial serum glucose level of 5.43 mmol/L (NR, 4.1-7.8mmol/L), and a normal HbA1_C_ level of 5.5% (NR, < 6.5%). However, a high homeostatic model assessment insulin resistance (HOMA-IR) score of 10.2 (NR, < 2.6) indicated insulin resistance (12). The sister of patient I.4 (1,II.1) was 9.8 years old. She was delivered at 40 weeks of gestation by caesarean section with normal birth weight of 3.3 Kg (-0.23 SDS). On physical examination, her phenotypic features were characteristic of Type A-IR, including acanthosis nigricans and severe hypertrichosis without obesity. Her height was 140.5 cm (+0.23 SDS) and her weight was 27 kg (-0.81 SDS) at the admission. Her pubertal development was Tanner stage II and her bone age was 10 years old. Her HbA1_C_ was elevated (7.1%). Oral glucose tolerance test results showed that she had diabetes mellitus with severe insulin resistance (HOMA-IR score, 25.1). Her testosterone level of 0.329 nmol/L was slightly elevated (prepubertal NR < 0.087 nmol/L; pubertal NR: <0.087-1.33 nmol/L). Pelvic ultrasound demonstrated normal size for age of both the uterus and the ovaries, without polycystic changes in both ovaries. Abdominal ultrasound was normal. Both parents were phenotypically normal and not obese. The father had normal fasting and postprandial blood glucose levels. An oral glucose tolerance test in the mother during the second pregnancy revealed normal glucose tolerance. Insulin levels were not determined.

The proband (2,II.1) of Kindred 2 was a 2-year-old Chinese boy, who was born as the first child to healthy, non-consanguineous parents (**Figure 1A**). He was delivered *via* cesarean section at 37 + 1 weeks of gestation because of severe intrauterine growth retardation. His birth weight, length, and head circumference were 1.6 kg (-3.33 SDS), 43 cm (-2.26 SDS), and 27cm (-4.36 SDS), respectively. At birth, he was noted to have dysmorphic characteristics typical of DS, including an elfin-like face, low-set large ears, upturned nostrils, thick lips, nipple hypertrophy, acanthosis nigricans, hirsutism, lack of subcutaneous fat, a distended abdomen and enlarged genitalia. Further examination revealed fluctuating blood glucose levels, ranging from 3-15 mmol/L, with fasting hypoglycemia and post-prandial hyperglycemia. His fasting serum insulin was high, at > 1000 mU/L (NR, 2.6-15 mU/L), as was his C-peptide level of > 13.33 nmol/L (NR, 0.3-1.5 nmol/L). Anti-insulin receptor antibodies were not detected. Serum electrolytes levels were in the normal range. Renin and aldosterone levels were not tested. Echocardiography demonstrated an atrial septal defect (7 mm). Abdominal ultrasound revealed nephrocalcinosis, with the liver, spleen, and bladder of normal size and structure. He failed to respond to exogenous insulin and his parents refused further treatment and blood glucose monitoring since he was 45 days old. During follow-up visits at an outpatient clinic, his HbA1_C_ level had increased to 9.5% at 5 months and 11.5% at 2 years. Global developmental delay and severe growth failure were noted. He sat unsupported at 10 months, walked independently at 2 years, and spoke his first words at 1.8 years. At 2 years of age, his height was 74.3 cm (-4.21 SDS), his weight was 8.4 kg (-3.6 SDS) and his head circumference was 44.9 cm (-2.77 SDS). The parents had normal phenotype and body mass index. Glucose tolerance tests performed in the parents revealed normal glucose tolerance but mild hyperinsulinemia (insulin at 0 and 120 minutes: 14.25 and 148.3 mU/L, 15.6 and 109.3 mU/L in the mother and father, respectively).

### Genetic Analysis

Whole-exome sequencing identified three missense variants (c.3670G>A, c.3614C>T and c.3355C>T) and one deletion variant (c.749_751del) in the *INSR* (NM_000208) gene in two kindreds (**Figure 1B**). Patients 1,II.1 and 1,II.2 from Kindred 1 were compound heterozygous for the novel G>A substitution at position 3670 c.3670G>A [p.(Val1224Met)] on the paternal allele and for the described variant c.3614C>T [p.(Pro1205Leu)] on the maternal allele. Patient 2,II.3 from Kindred 2 was compound heterozygous for a novel 3-bp deletion resulting in the deletion of Thr at position 250 c.749_751del [p.(Thr250del)] on the maternal allele; and a known missense variant c.3355C>T [p.(Arg1119Trp)] on the paternal allele. Thr250 and Val1224 are located in the CR and TK domains of INSR, respectively, and the valine at codon 1224 is highly conserved among different species (**Figure 1C**).

*In silico* analysis with Mutation Taster and Provean predicted the effect of the deletion variant p.Thr250del as “disease causing” or “deleterious”, respectively. The missense variant p.Val1224Met was predicted to be highly damaging to protein function (SIFT: 0, PolyPhen2: 1, Mutation Taster: “disease causing”, Provean: “deleterious”).

### Cellular Localization of WT and Mutated INSR in CHO Cells

**Figure 2:**
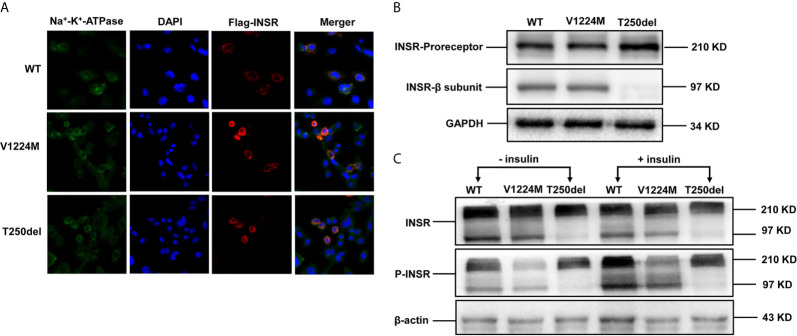
Expression and processing of wild-type (WT) and mutant INSRs in Chinese hamster ovary (CHO) cells. **(A)** Immunofluorescence of CHO cells transiently transfected with the indicated FLAG-tagged INSR, WT or mutant vectors (p.V1224M and p.T250del). **(B)** Western blot analysis of the INSR proreceptor (210 kD) and the mature β-submit of the INSR (97 kD). **(C)** Insulin-stimulated phosphorylation of INSR and western blot analysis of INSR phosphorylation. DAPI, 4’,6-diamidino-2-phenylindole.

The mutant INSRs were predominantly co-localized with the Na^+^/K^+^-ATPase (a plasma membrane marker), similar to the WT protein, suggesting that the cellular membrane localization of the mutated form of INSR was not impaired in either case (**Figure 2A**).

### Expression of INSR/p-INSR and Akt/p-Akt Proteins in CHO Cells

**Figure 3:**
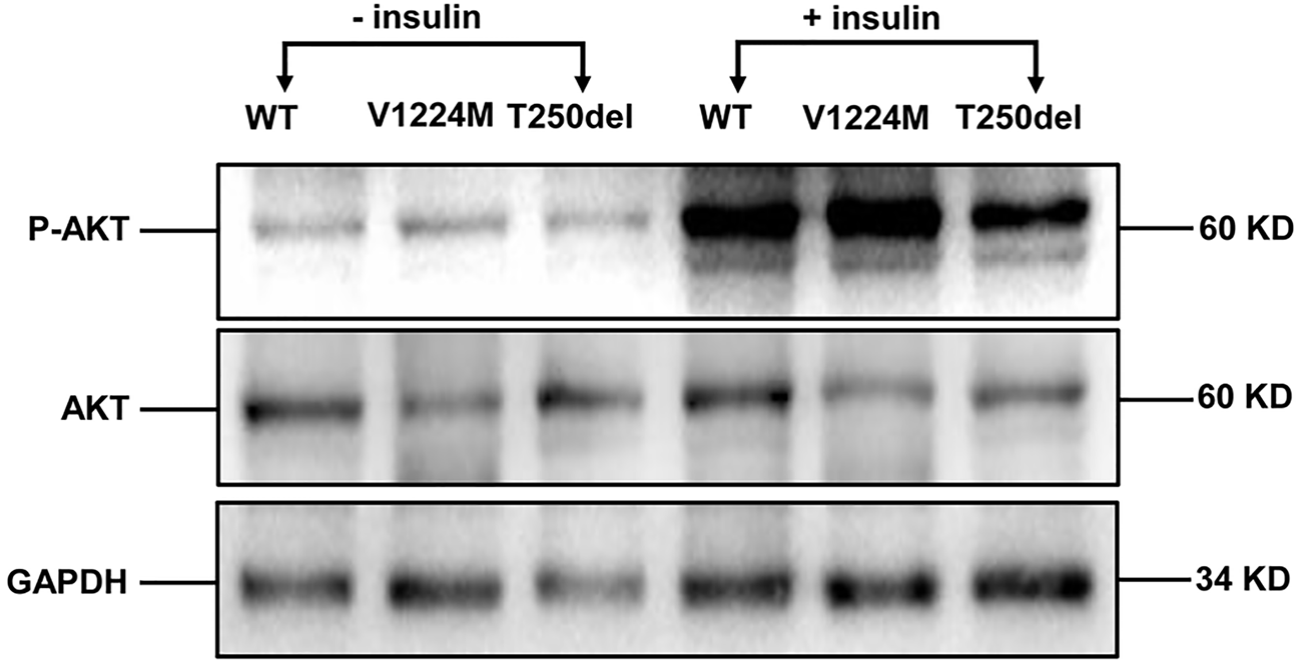
Insulin-stimulated phosphorylation of Akt in CHO cells expressing WT and mutant INSRs. Western blot analysis of the total Akt (60 kD) and phospho-Akt (60 kD) with and without insulin stimulation.

Western blot analysis showed that expression of the INSR proreceptor in the two mutants was not lower than that in WT (**Figure 2B**). In contrast, the levels of mature INSR (β-subunit) were lower than WT in cells expressing the Thr250del mutant, but not in cells expressing the Val1224Met mutant. This suggests an impairment in proreceptor processing in the Thr250del mutant (**Figure 2B**). Moreover, the phosphorylation of INSR and Akt was shown to be decreased in CHO cells transfected with Thr250del mutant, as compared to WT INSR (**Figures 2C** and **3**). However, the phosphorylation of INSR and Akt was not impaired by the Val1224Met mutant compared with WT INSR (**Figures 2C** and **3**).

## Discussion

Monogenic causes of insulin resistance are usually severe and associated with rare inherited disorders resulting from mutations in the insulin signaling pathway (13). Numerous genes are candidates for severe insulin resistance, such as INSR, BSCL2, AGPAT2, CAV1, and PTRF (14). Variants in INSR result in rare severe insulin resistance syndromes, including DS, RMS and Type A-IR. In the present study, we describe three patients from two kindreds with Type A-IR and DS. Four variants of the *INSR* gene were identified, including two novel variants (c.3670G>A and c.749_751del).

Patients 1,II.1 and 1,II.2 from Kindred 1 with type A-IR, carried the compound heterozygous missense variants c.3670G>A [p.(Val1224Met)] and c.3614C>T [p.(Pro1205Leu)]. Generally, patients with type A-IR have heterozygous variants in the β-subunit. Some patients with type A-IR have also been reported to have biallelic variants (homozygous or compound heterozygous) (15–17). The residue Pro1205 is a highly-conserved amino acid in the TK domain and located near autophosphorylation site, so this variant was thought to impair insulin receptor function. The heterozygous variant p.Pro1205Leu was previously described in a 14-year-old Japanese female patient with obesity, moderate insulin resistance, acanthosis nigricans, hypertrichosis and hyperandrogenism (18). Two other British female patients who were both heterozygous for this variant presented with acanthosis nigricans, hirsutism, and asymptomatic diabetes but without obesity (19, 20). It can be seen that obesity can be a phenotypic feature of patients with type A-IR. The novel variant c.3670G>A caused the replacement of a highly-conserved valine residue with a methionine in position 1224 of the TK domain (**Figure 1C**). The previously-reported variants near Val1224, such as c.3659G>T [p.(Trp1220Leu)] and c.3680G>C [p.(Trp1227Ser)] caused type A-IR in a dominant manner. These variants were further found to cause defective tyrosine kinase activity of the INSR in an *in vitro* autophosphorylation assay (21, 22). In contrast, the receptor expression and insulin-induced phosphorylation of INSR and Akt in cells with the transfection of Val1224Met mutant INSR were largely normal compared to the WT control. These results suggest that the variant p.Val1224Met appears to be tolerated and the limitation of bioinformatics analyses in predicting the pathogenicity of variants. Notably, the two siblings from Kindred 1 carrying the identical *INSR* variants showed normal height and short stature, respectively. Similar to the reported patients with type A-IR, members of the same kindred had different growth pattern (4), which may be related to other genetic or environmental influence.

Patient 2,II.1 presented with features typical of DS. Less common clinical features (atrial septal defect and nephrocalcinosis) were also identified and have been reported in other patients with DS (23, 24). Patients with severe disorders (DS or RMS) are usually homozygous or compound heterozygous for *INSR* variants in the α-subunit. Patient 2,II.1 was compound heterozygous for the novel variant c.749_751del [p.(Thr250del)] and known variant c.3355C>T [p.(Arg1119Trp)], located in the α- and β-subunits, respectively. The p.Arg1119Trp variant was previously identified in patients with RMS (compound heterozygous with a 2.3kb microdeletion covering part of exon 10 and all of exon 11) and DS (compound heterozygous with p.Glu1206Lys in the β-subunit) (2, 25). Another variant c.3556G>A [p.(Arg1119Gln)] at the same amino acid position was previously described in a homozygous patient with DS (26). Variants at codon 1119 have previously been reported to cause a loss of tyrosine kinase activity of the INSR without affecting insulin binding (15, 26). Thr250 is in a highly conserved part of the extracellular CR domain of the INSR protein and located in a C229–C252 disulfide bond which is essential to maintain the dimensional structure of the INSR protein. Therefore, the Thr250del variant may interfere with the WT disulfide bonding pattern, and thus affect the stability and local geometry of the insulin receptor. In close proximity to our p.Thr250del variant, a missense variant at amino acid position 260 (p.Leu260Pro) and 256 (p.Arg256Cys) were shown to completely block processing and transport of the insulin receptor to the cell surface in vitro (27). In our study, the functional analysis also showed that the Thr250del variant was associated with reduced expression of the mature INSR and impaired processing of the proreceptor form. In addition, the severely impaired phosphorylation was mainly due to decreased expression of mature receptors caused by the Thr250del variant. Taken together, the functional characterization of the Thr250del variant was consistent with its severe phenotype of DS. Notably, the mother and father of the proband, an obligate heterozygote for the variant p.(Thr250del) and p.(Arg1119Trp), respectively, were phenotypically normal but had mild insulin resistance. Thus, although the DS is inherited as an autosomal recessive trait, the phenotype of insulin resistance may be inherited in a codominant fashion.

In summary, we have described in detail the phenotype of three patients with *INSR*-related insulin resistance syndrome. DS was attributable to compound heterozygotes with two variants in the a- and β-subunits. Further, the novel Thr250del variant was shown to impair proreceptor processing. The other two siblings with type A-IR were compound heterozygotes with two missense variants (p.Val1224Met, p.Pro1205Leu) in the TK region of the β-subunit. The novel variant Val1224Met showed normal expression of the mature INSR and INSR activity in CHO cells *in vitro*, which suggested this variant was not responsible for the severe insulin resistance. The three disorders caused by defects in the INSR should be considered as a continuous spectrum, which are diagnosed based on characteristic clinical features and identification of *INSR* pathogenic variants on molecular genetic analysis. The patients with *INSR*-related insulin resistance syndrome manifest with distinct phenotypic and genotypic heterogeneity, which may depend on the genetic or environmental background.

## Data Availability Statement

The datasets generated for this study can be found in the LOVD database *via* the following links: https://databases.lovd.nl/shared/screenings/0000360822 and https://databases.lovd.nl/shared/screenings/0000360820.

## Ethics Statement

The studies involving human participants were reviewed and approved by the ethics committee of the Children’s Hospital of Nanjing Medical University (Nanjing, China). Written informed consent to participate in this study was provided by the participants’ legal guardian/next of kin.

## Author Contributions

QZ and JY are the co-first authors. QZ and JY collected samples, performed the experimentation, and analyzed the data. AZ and WG conceived and designed this study. XY and ZZ collected the clinical samples and clinical data. CW performed whole-exome sequencing analysis. WG, AZ, QZ and YJ wrote the manuscript. All authors contributed to the article and approved the submitted version.

## Conflict of Interest

The authors declare that the research was conducted in the absence of any commercial or financial relationships that could be construed as a potential conflict of interest.
