# Supplementary material for: Clinical and Functional Characterization of Novel INSR Variants in Two Families With Severe Insulin Resistance Syndrome
Source: Front Endocrinol (Lausanne). 2021 Apr 29;12:606964. doi: 10.3389/fendo.2021.606964 (PMC8117416; doi:10.3389/fendo.2021.606964)
Supplement: Supplementary file 1 [file DataSheet_1.docx]

**Patient Perspective (Patient 1,II.1)**She is an outgoing girl. She likes drawing and dancing. She was very confused and scared, when she was diagnosed with insulin resistance and diabetes. She began to understand the mechanism and characteristics of this disease with the help of the endocrinologist and geneticist. She feels fine now. In order to control her blood sugar level, she needs a proper diet and exercise, blood sugar monitoring and oral metformin treatment.
